# Acute respiratory distress syndrome: does histology matter?

**DOI:** 10.1186/s13054-015-1022-6

**Published:** 2015-09-15

**Authors:** José A. Lorente, Aida Ballén-Barragán, Raquel Herrero, Andrés Esteban

**Affiliations:** Hospital Universitario de Getafe, Carretera de Toledo km 12500, 28905 Madrid, Spain; CIBER de Enfermedades Respiratorias, Instituto de Salud Carlos III, Calle Sinesio Delgado, 4, 28029 Madrid, Spain; Universidad Europea de Madrid, Calle Tajo, s/n, 28670 Villaviciosa de Odón, Madrid Spain

## Abstract

Kao et al. have reported in *Critical Care* the histological findings of 101 patients with acute respiratory distress syndrome (ARDS) undergoing open lung biopsy. Diffuse alveolar damage (DAD), the histological hallmark of ARDS, was present in only 56.4 % of cases. The presence of DAD was associated with higher mortality. Evidence from this and other studies indicates that the clinical criteria for the diagnosis of ARDS identify DAD in only about half of the cases. On the contrary, there is evidence that the clinical course and outcome of ARDS differs in patients with DAD and in patients without DAD. The discovery of biomarkers for the physiological (increased alveolocapillary permeability) or histological (DAD) hallmarks of ARDS is thus of paramount importance.

Kao et al. [1] have made an important contribution to the knowledge of the histological changes associated with acute respiratory distress syndrome (ARDS) and the potential role of open lung biopsy (OLB) in the diagnosis and management of ARDS. The authors studied the histological findings in OLB from 101 patients with a diagnosis of ARDS over 15 years. Indications for OLB included a suspicion of noninfectious cause that could benefit from corticosteroid treatment. Notwithstanding the obvious selection bias, histological information from OLB or autopsy tissue samples is of cardinal importance for a better understanding of the pathogenesis and management of ARDS. Kao et al.’s [1] main findings are that diffuse alveolar damage (DAD) was present in only 56.4 % of patients with ARDS, and that the presence of DAD was associated with a worse outcome in patients with ARDS. The results of OLB, in accordance with other studies [2–5], changed management in a substantial proportion of patients.

ARDS is a syndrome of acute respiratory failure due to pulmonary inflammation developing after a known risk factor, leading to increased endothelial and epithelial permeability, pulmonary edema, hypoxemia, loss of aerated tissue, decreased lung compliance, and bilateral opacities in the chest X-ray image. The histological correlate of ARDS is DAD, characterized by lung edema, inflammation, hemorrhage, hyaline membranes, and alveolar epithelial cell injury [6–8].

The agreement between the clinical diagnosis of ARDS according to commonly accepted criteria [7, 8] and the presence of DAD at histological examination is poor, ranging from 13 to 58 % in studies using OLB [2–5, 9, 10] and from 45 to 88 % in autopsy studies [11–16]. In two autopsy studies using the Berlin definition of ARDS [8], DAD was present in only 45 % of patients diagnosed with ARDS [11, 12].

Conditions identified in patients without DAD include, among others, organizing pneumonia, eosinophilic pneumonia, pulmonary embolism, drug-induced pneumonitis, alveolar hemorrhage, lymphangitis, malignancy, or vasculitis. Of note, in one study [12] 14 % of patients with ARDS did not have pathological changes, probably representing cases with diffuse atelectasis that appear clinically as ARDS but resolve as the lungs are inflated at high pressure prior to fixation.

Many of the conditions identified at the histological examination do not share the same pathogenesis, treatment, and biomarkers as DAD. The failure of previous studies into the treatment of ARDS has thus been attributed in part to a lack of a reliable definition of ARDS designating a homogeneous phenotype.

The importance of identifying a homogeneous phenotype in ARDS is highlighted by the finding in the study by Kao et al. [1] of different mortality rates in patients with DAD and in patients without DAD (71.9 % versus 41.5 %). In a recent study, Guerin et al. [9] reported in 83 patients with ARDS undergoing OLB a higher airway plateau pressure, worse oxygenation, and (not reaching statistical significance) higher mortality in patients with DAD versus patients without DAD. These findings [1, 9] together suggest that the histological finding of DAD defines a specific population of patients within the syndrome of ARDS.

The heterogeneity of conditions designated with the same clinical diagnostic criteria as well as data suggesting that the clinical course differs in patients with DAD and in patients without DAD thus underline the importance of identifying patients with specific clinicopathological phenotypes within those diagnosed with ARDS.

Another challenge for our understanding of ARDS is the identification of the mechanisms explaining why some patients with a clinical risk factor go on to develop ARDS whereas others do not. Hopefully, the recognition of these patients at risk should be accomplished early in their course, before the requirement of ventilatory support. Biomarkers should thus be determined in the blood rather than in bronchoalveloar lavage fluid.

In conclusion, after almost five decades of research [6], ARDS continues to pose challenges for physicians and scientists. The discovery of markers for the physiological (e.g., alveolocapillary hyperpermeability) or histological (hyaline membrane) hallmarks of ARDS is of great import for the identification of a specific phenotype within ARDS.

## Abbreviations

ARDS: Acute respiratory distress syndrome
DAD: Diffuse alveolar damage
OLB: Open lung biopsy
